# Supplementary material for: Disability services in higher education: Statistical disparities and the potential role of AI in bridging institutional gaps
Source: PLoS One. 2025 May 7;20(5):e0322728. doi: 10.1371/journal.pone.0322728 (PMC12057937; doi:10.1371/journal.pone.0322728)
Supplement: S1 Appendix — (DOCX) [file pone.0322728.s001.docx]

Appendix A: Detailed Graduation, Enrollment and Employment Data for Adults with Disabilities

| **State** | **High School Completion Rate (%)** | **2-Year Higher Education Enrollment (%)** | **4-Year Higher Education Enrollment (%)** | **Adults Neither Enrolled Nor Working (%)** |
| --- | --- | --- | --- | --- |
| Alabama | 90 | 15 | 31 | 13 |
| Alaska | 78 | 12 | 28 | 14 |
| Arizona | 80 | 18 | 32 | 12 |
| Arkansas | 82 | 20 | 29 | 11 |
| California | 84 | 30 | 35 | 15 |
| Colorado | 85 | 22 | 32 | 13 |
| Connecticut | 89 | 16 | 38 | 14 |
| Delaware | 87 | 20 | 34 | 12 |
| Florida | 85 | 29 | 37 | 14 |
| Georgia | 86 | 25 | 35 | 13 |
| Hawaii | 90 | 18 | 33 | 11 |
| Idaho | 83 | 15 | 30 | 14 |
| Illinois | 87 | 25 | 36 | 13 |
| Indiana | 88 | 19 | 34 | 12 |
| Iowa | 91 | 22 | 35 | 12 |
| Kansas | 89 | 20 | 36 | 14 |
| Kentucky | 84 | 19 | 30 | 15 |
| Louisiana | 80 | 16 | 29 | 14 |
| Maine | 89 | 25 | 32 | 13 |
| Maryland | 88 | 28 | 39 | 14 |
| Massachusetts | 89 | 21 | 34 | 12 |
| Michigan | 88 | 26 | 36 | 13 |
| Minnesota | 91 | 18 | 37 | 13 |
| Mississippi | 85 | 19 | 30 | 15 |
| Missouri | 88 | 21 | 33 | 14 |
| Montana | 84 | 16 | 31 | 12 |
| Nebraska | 87 | 19 | 29 | 13 |
| Nevada | 82 | 16 | 32 | 14 |
| New Hampshire | 92 | 28 | 40 | 13 |
| New Jersey | 91 | 25 | 35 | 12 |
| New Mexico | 90 | 26 | 38 | 13 |
| New York | 89 | 21 | 33 | 14 |
| North Carolina | 91 | 20 | 35 | 14 |
| North Dakota | 89 | 22 | 33 | 13 |
| Ohio | 88 | 19 | 36 | 14 |
| Oklahoma | 86 | 15 | 30 | 15 |
| Oregon | 88 | 26 | 37 | 12 |
| Pennsylvania | 89 | 22 | 35 | 13 |
| Rhode Island | 85 | 20 | 32 | 12 |
| South Carolina | 82 | 16 | 31 | 11 |
| South Dakota | 85 | 19 | 33 | 13 |
| Tennessee | 91 | 22 | 36 | 12 |
| Texas | 89 | 20 | 35 | 11 |
| Utah | 90 | 19 | 32 | 13 |
| Vermont | 91 | 26 | 34 | 12 |
| Virginia | 90 | 25 | 37 | 13 |
| Washington | 91 | 22 | 33 | 12 |
| West Virginia | 92 | 25 | 34 | 14 |
| Wisconsin | 85 | 18 | 30 | 13 |
| Wyoming | 84 | 17 | 31 | 12 |

**Note:** Data sourced from the National Center for Education Statistics (NCES, 2023) and state-level education reports from the U.S. Department of Education (2010), and U.S. Department of Labor (2022).
